# Evidence of a DHA Signature in the Lipidome and Metabolome of Human Hepatocytes

**DOI:** 10.3390/ijms18020359

**Published:** 2017-02-08

**Authors:** Veronica Ghini, Mattia Di Nunzio, Leonardo Tenori, Veronica Valli, Francesca Danesi, Francesco Capozzi, Claudio Luchinat, Alessandra Bordoni

**Affiliations:** 1 Center of Magnetic Resonance (CERM), University of Florence, Via Luigi Sacconi, 6-50019 Sesto Fiorentino (FI), Italy; ghini@cerm.unifi.it (V.G.); luchinat@giottobiotech.com (C.L.); 2 Interdepartmental Centre for Industrial Agri-Food Research, University of Bologna, Via Quinto Bucci, 336-47521 Cesena (FC), Italy; mattia.dinunzio@unibo.it (M.D.N.); francesco.capozzi@unibo.it (F.C.); 3 Department of Experimental and Clinical Medicine, University of Florence, Largo Brambilla, 3-50134 Florence (FI), Italy; tenori@cerm.unifi.it; 4 Department of Agri-Food Sciences and Technologies (DISTAL), University of Bologna, Piazza Goidanich, 60-47521 Cesena (FC), Italy; veronica.valli9@unibo.it (V.V.); francesca.danesi@unibo.it (F.D.); 5 Department of Chemistry, University of Florence, Via della Lastruccia, 3-50019 Sesto Fiorentino (FI), Italy; 6 GIOTTO Biotech S.r.l., Via Madonna del Piano, 6-50019 Sesto Fiorentino (FI), Italy

**Keywords:** docosahexaenoic acid, propionic acid, protocatechuic acid, nuclear magnetic resonance (NMR), metabolomics, lipidomics, hepatocytes

## Abstract

Cell supplementation with bioactive molecules often causes a perturbation in the whole intracellular environment. Omics techniques can be applied for the assessment of this perturbation. In this study, the overall effect of docosahexaenoic acid (DHA) supplementation on cultured human hepatocyte lipidome and metabolome has been investigated using nuclear magnetic resonance (NMR) in combination with traditional techniques. The effect of two additional bioactives sharing with DHA the lipid-lowering effect—propionic acid (PRO) and protocatechuic acid (PCA)—has also been evaluated in the context of possible synergism. NMR analysis of the cell lipid extracts showed that DHA supplementation, alone or in combination with PCA or PRO, strongly altered the cell lipid profile. The perfect discrimination between cells receiving DHA (alone or in combination) and the other cells reinforced the idea of a global rearrangement of the lipid environment induced by DHA. Notably, gas chromatography and fluorimetric analyses confirmed the strong discrimination obtained by NMR. The DHA signature was evidenced not only in the cell lipidome, but also in the metabolome. Results reported herein indicate that NMR, combined with other techniques, represents a fundamental approach to studying the effect of bioactive supplementation, particularly in the case of molecules with a broad spectrum of mechanisms of action.

## 1. Introduction

Proper nutrition offers one of the most effective and least costly ways to decrease the burden of many diet-related diseases (DRD) and their associated risk factors. In this context, food bioactive compounds are considered a promising tool that needs further exploitation. Notwithstanding, reliable, science-based information on the health benefits of bioactive foods and constituents is lacking [1].

Although the highest quality of evidence is the randomized controlled trial (RCT) [2], this design is often very expensive and impractical for the long latency effects observed for many diet-disease links. In addition, in RCT, many confounding factors can lead to contradictory results. Moreover, an RCT cannot be ethically designed to evaluate the benefit of the presence of nutrients (and staple foods) that are naturally present in the diet compared with their absence. As a result, the number of bioactive components with regulatory approval by the European Food Safety Authority (EFSA) or the Food and Drug Administration (FDA) is low.

Cultured cells represent a controlled and defined model system that could help demonstrating the causality for bioactive compounds and health, which is one of the mandatory requirements to get regulatory approval of health claims. Nevertheless, in vitro studies often lack a comprehensive system-level vision. In fact, it is seldom considered that cell supplementation with a bioactive molecule has an impact not only on specific targets (namely the endpoints of the study), but often causes a perturbation in the whole intracellular environment.

In this study, the overall effect of docosahexaenoic acid (DHA) supplementation on human hepatocyte lipidome and metabolome has been simultaneously investigated using NMR-based metabolomics. Traditional techniques such as gas chromatography (GC) have been used to validate NMR results on the lipidome.

DHA has been reported to be beneficial on various disease endpoints [3], although its mechanism of action is still largely unknown. In vitro studies have revealed that DHA is taken up by cells [4], and it is directly incorporated into membrane fractions that are detergent-resistant and roughly correspond to rafts [5,6]. The DHA-containing phospholipids (PL) are substantially more disordered than their more common counterparts [7], and they have poor affinity for cholesterol [8]. n-3 long-chain polyunsaturated fatty acids (n-3 LC-PUFA) displace cholesterol from rafts, and internalize it into the cell. As a consequence, a significant change in the organization of the plasma membrane and signaling proteins is routinely observed in cells after DHA supplementation [6,9].

DHA supplementation also impacts cell homeostasis through other mechanisms, i.e., the generation of specialized pro-resolving lipid mediators and the modulation of gene expression [10,11]. Overall, a deep perturbation in the genome, lipidome, proteome and metabolome is supposed to take place after DHA supplementation. Omics techniques can be applied for the assessment of this perturbation. Different studies have been performed to evaluate DHA-induced modification in the global gene expression using microarrays [12,13,14]. Moreover, the regulation of eicosanoids by n-3 LC-PUFA has been studied using liquid chromatography-tandem mass spectrometry (LC-MS/MS)-based targeted metabolomics [15]. In addition, solid state ^2^H nuclear magnetic resonance (NMR) spectroscopy of deuterated model bilayers has been used to elucidate underlying mechanisms by which n-3 LC-PUFA-containing phospholipids can regulate molecular organization of lipid micro-domains [16]. Although all these studies have provided important information, the vision of the effects of DHA on cells is still fragmented, and a “foodomics” vision connecting food bioactives and cell behavior is needed [17].

Data reported herein indicate that NMR can be successfully applied to simultaneously investigate perturbations in cell lipidome and metabolome not only after DHA supplementation, but also after other non-lipid molecules. In fact, in this study, NMR has been used to evaluate the effects of other bioactives, namely propionic acid (PRO) and protocatechuic acid (PCA), that have been reported to share with DHA the blood lipid-lowering effect [18,19,20]. In addition, the effect of the combined supplementation of DHA with PRO or PCA has been verified.

## 2. Results

To test with a fast and untargeted approach the overall effects of supplementation of DHA, PCA and PRO on hepatic cell lipidome and metabolome, NMR-based metabolomic analysis of both lipid and water extracts was used, combined with GC and fluorimetric-based lipid analysis. Six groups of HepG2 human hepatoma cells were analyzed: (i) not supplemented cells (NS); (ii) cells supplemented with DHA alone (DHA); (iii) cells supplemented with DHA in combination with PRO (DHA + PRO); (iv) cells supplemented with DHA in combination with PCA (DHA + PCA); (v) cells supplemented with PRO alone (PRO); (vi) cells supplemented with PCA alone (PCA).

Cultured liver cells have been used as a model system since they are important players in both extra-hepatic and hepatic lipid metabolism.

DHA uptake is time-dependent, and DHA effects largely depend on its concentration within the cell; therefore, all analyses were performed after 6 and 24 h of supplementation. Since preliminary experiments evidenced that DHA uptake is higher after 24 than 48 h supplementation (data not shown), the “peak” effect of DHA was expected at the 24 h time point.

### 2.1. Lipidome

**Figure 1:**
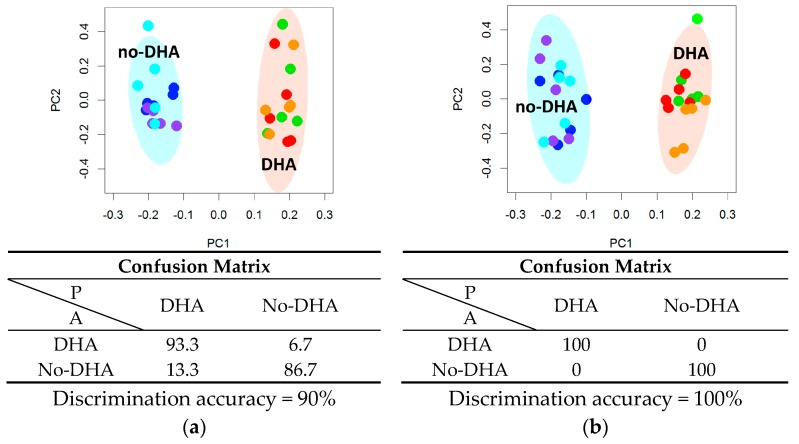
Lipidomic phenotyping by nuclear magnetic resonance (NMR) analysis of cells not supplemented and supplemented with docosahexaenoic acid (DHA), alone or in combination with protocatechuic acid (PCA) or propionic acid (PRO), for (**a**) 6 h and (**b**) 24 h. Principal component analysis and canonical analysis (PCA-CA) score plots: PC1 vs. PC2. In each group, five samples derived from three independent experiments were analyzed. Each symbol represents a different sample. Blue symbols = NS; purple symbols = PCA; cyan symbols = PRO; orange symbols = DHA; green symbols = DHA + PCA; red symbols = DHA + PRO. For each PCA-CA model, the confusion matrix and the discrimination accuracy are reported; A: actual classes; P: predicted classes.

NMR analysis of the lipid extracts evidenced that DHA supplementation, alone or in combination with PCA or PRO, strongly altered the cell lipid profile. Resonances attributable to DHA were observed in the spectra (Table S1) of the lipid extracts, indicating that DHA is already absorbed after 6 h supplementation. These signals (buckets) were not included in the following multivariate analysis to avoid the trivial separation due to the addition of DHA in the samples, thus underlining the changes in the global lipidome profile. Multivariate statistical analysis (principal component analysis and canonical analysis, PCA-CA) showed a clear-cut discrimination between cells supplemented with DHA (DHA, DHA + PCA, DHA + PRO) and cells not supplemented with DHA (NS, PCA, PRO), indicated as “DHA” group and “no-DHA” group respectively, with a cross-validated accuracy of 90% at 6 h, and of 100% at 24 h (Figure 1 and Figure S1). On the contrary, supplementation with PRO and PCA had no detectable effects on HepG2 global lipid profile (Tables S2 and S3).

From the PC1 loading plots (Figure S1) it is possible to infer the spectral regions mostly contributing to the separation between “DHA” and “no-DHA” groups, both at 6 and 24 h. In particular, a decrement of total cholesterol, saturated fatty acids (SFA) and monounsaturated fatty acids (MUFA) as well as an increment of triglycerides (TG) was observed. Univariate statistical analysis on the identified and assigned lipid group in the NMR spectra (Table S1) was also performed, and results are shown in Table 1.

After 6 and 24 h supplementation, the fatty acid composition of not supplemented (NS) and supplemented cells was also evaluated by GC analysis (Table 2 and Table 3). GC analysis mostly confirmed NMR data. In fact, according to signals in the NMR spectra, after both 6 and 24 h supplementation, significant increases in the concentration of DHA, the unsaturation index (UI), n-3/n-6 ratio and total PUFA content were observed in cells of the “DHA” group compared to the “no-DHA” one (Figure S2). On the contrary, after 6 h, the fatty acid profile was not modified in cells supplemented with PRO and PCA alone (Table 2).

After 24 h (Table 3) all DHA-supplemented cells showed a decrease in linoleic acid content, as already evidenced by NMR. At this time point, a significant reduction in the content of the main saturated fatty acids and of linoleic acid (18:2 n-6), as well as a significant decrease in the total fatty acid content, was detected in PRO- and PCA-supplemented cells.

**Figure 2:**
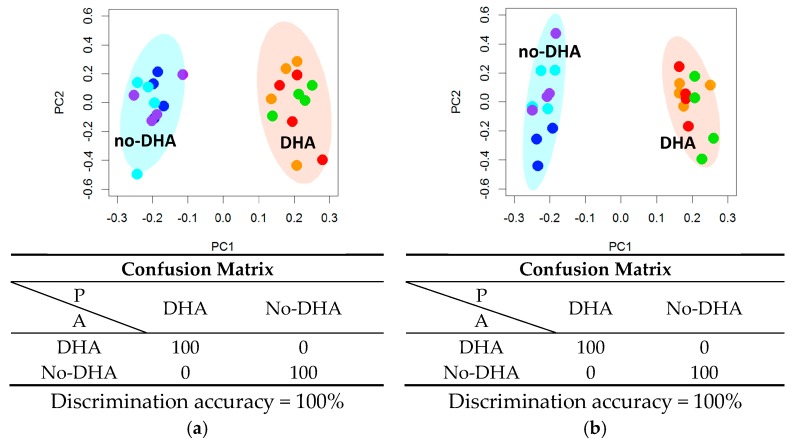
Fatty acid phenotyping by gas chromatography (GC) analysis of cells not supplemented (NS) and supplemented with docosahexaenoic acid (DHA), alone or in combination with protocatechuic acid (PCA) or propionic acid (PRO), after (**a**) 6 h and (**b**) 24 h supplementation. Principal component analysis and canonical analysis (PCA-CA) score plots: PC1 vs. PC2. In each group, four samples derived from three independent experiments were analyzed. Each symbol represents a different sample. Blue symbols = NS; purple symbols = PCA; cyan symbols = PRO; orange symbols = DHA; green symbols = DHA + PCA; red symbols = DHA + PRO. For each PCA-CA model, the confusion matrix and the discrimination accuracy are reported; A: actual classes; P: predicted classes.

A PCA-CA analysis was performed on the GC fatty acid composition data, showing a perfect separation on the PC1 axis, between “DHA” and “no-DHA” groups (Figure 2), with a discrimination accuracy of 100% for both 6 h and 24 h data sets. The corresponding PC1 loading plots (Figure S3) highlight the increase of DHA concentration, UI, n-3/n-6 ratio, and total PUFA content in the “DHA” group. Despite the differences in the fatty acid composition observed by GC, PCA-CA analysis did not allow discrimination based on supplementation with PRO or PCA (Tables S4 and S5).

**Figure 3:**
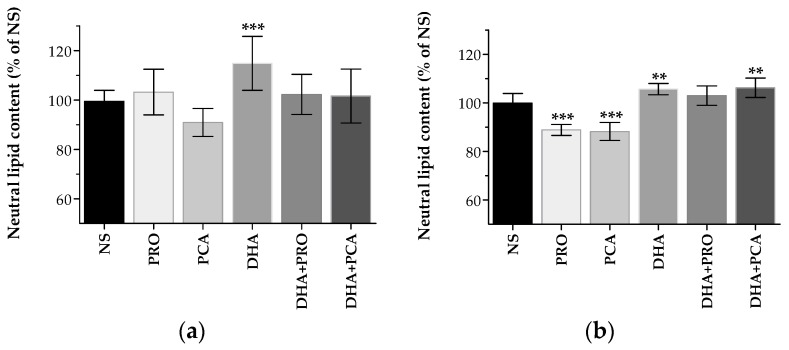
Lipid droplet accumulation in not supplemented (NS) and supplemented cells with docosahexaenoic acid (DHA), protocatechuic acid (PCA) and propionic acid (PRO), after (**a**) 6 h and (**b**) 24 h supplementation. Data in each group are means ± standard deviation (SD) of five samples derived from two independent experiments. Neutral lipid content is expressed as the percentage of the value obtained in NS cells (assigned as 100%). Statistical analysis was carried out by one-way ANOVA (panels (**a**,**b**) *p* < 0.001) using Dunnett’s post-test to compare NS and supplemented cells (** *p* < 0.01; *** *p* < 0.001).

Oil Red O staining, which allows detecting neutral lipids [21] was used to evaluate lipid droplet (LD) accumulation in supplemented cells. At 6 h, a significant increase in neutral lipids was evidenced in cells supplemented with DHA, and at 24 h in cells supplemented with DHA and DHA + PCA (Figure 3). These data are in agreement with the increased TG signals in the spectra of “DHA” group compared to “no-DHA” one.

**Figure 4:**
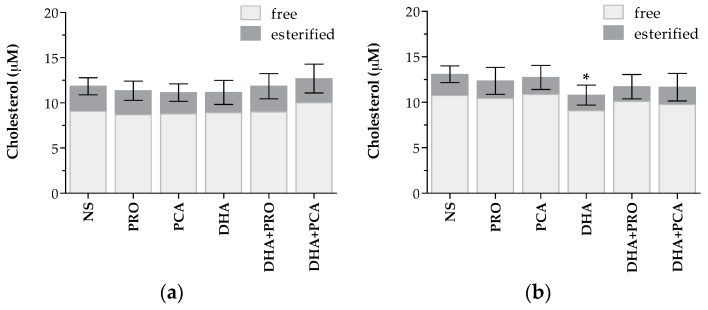
Intracellular cholesterol concentration in not supplemented (NS) and supplemented cells with docosahexaenoic acid (DHA), protocatechuic acid (PCA) and propionic acid (PRO) after (**a**) 6 h and (**b**) 24 h supplementation. Data are expressed in µM cholesterol per well, and are means ± standard deviation (SD) of five samples derived from three independent experiments. Statistical analysis was carried out by the one-way ANOVA (panels (**a**,**b**) *p* < 0.05) using Dunnett’s post-test to compare NS and supplemented cells (* *p* < 0.05).

No differences in total, free or esterified cholesterol concentration were detected after 6 h supplementation (Figure 4A). However, after 24 h, a significant decrease in the total cholesterol content was detected in cells supplemented with DHA alone (Figure 4B). This decrease was mainly due to a reduction in free cholesterol content (*p* < 0.05). The combined supplementation of DHA + PRO and DHA + PCA also caused a trend to decrease cell cholesterol content, although it was not significant compared to NS cells. In agreement, a significant reduction of signals assigned to total and free cholesterol was observed in NMR spectra of cells of the “DHA” group compared to “no DHA” after 24 h supplementation (Table 1, Figure S4).

### 2.2. Metabolome

NMR-based metabolomic analysis of water extracts was used to analyze the overall metabolomic effect of cell supplementation.

**Figure 5:**
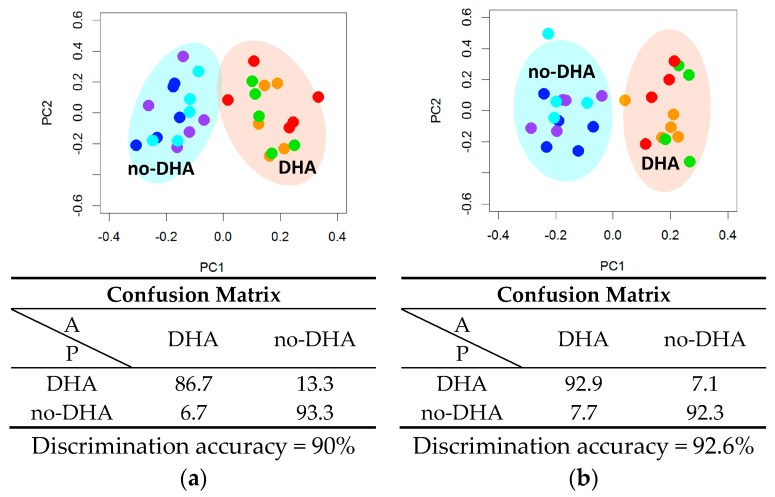
Cytoplasmic metabolomic phenotyping by nuclear magnetic resonance (NMR) analysis of cells not supplemented and supplemented cells with docosahexaenoic acid (DHA), alone or in combination with protocatechuic acid (PCA) or propionic acid (PRO), after (**a**) 6 h and (**b**) 24 h supplementation. Principal component analysis and canonical analysis (PCA-CA) score plots: PC1 vs. PC2. In each group, five samples derived from three independent experiments were analyzed. Each symbol represents a different sample. Blue symbols = NS; purple symbols = PCA; cyan symbols = PRO; orange symbols = DHA; green symbols = DHA + PCA; red symbols = DHA + PRO. For each PCA-CA model, the confusion matrix and the discrimination accuracy are reported; A: actual classes; P: predicted classes.

**Figure 6:**
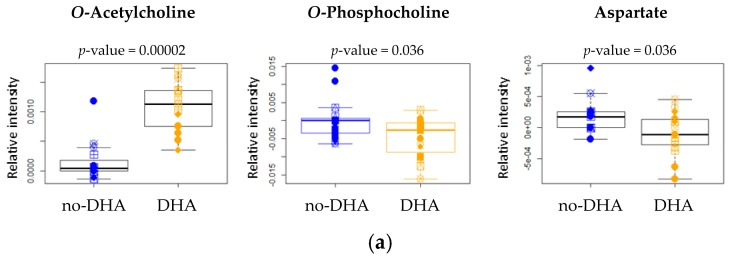

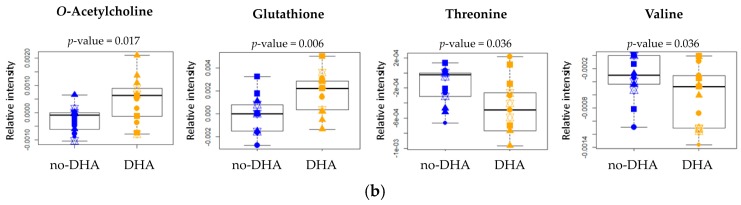
Box plots of the metabolites differentially concentrated in “no-DHA” (not supplemented (NS), protocatechuic acid (PCA), propionic acid (PRO), blue symbols) and “DHA” group (docosahexaenoic acid (DHA), DHA + PCA, DHA + PRO, yellow symbols) according to NMR analysis after (**a**) 6 h and (**b**) 24 h supplementation. For each comparison, the *p*-value (by the Wilcoxon test) is also reported.

The same multivariate untargeted approach used to analyze lipid extracts showed that DHA supplementation, alone or in combination with PCA and PRO, was sufficient to radically alter also the cytoplasmic metabolome (Figure 5 and Figure S5). Using PCA-CA statistical analysis, a discrimination accuracy of 90% was obtained for the comparison between the “no-DHA” and “DHA” groups after 6 h supplementation. In particular, the “DHA” group was characterized by significantly higher levels of *O*-acetylcholine and slight but significant decrements of *O*-phosphocholine and aspartate (Figure 6 and Table 4).

The discrimination accuracy increases up to 93%, when considering cells collected after 24 h supplementation (Figure 5 and Figure S5). After 24 h, *O*-acetylcholine level was still higher in the “DHA” group than in the “no-DHA” one (Figure 6 and Table 4). Moreover, the metabolic profile of the “DHA” group showed a strong increment of glutathione (GSH) levels, and a weak decrement of threonine and valine levels with respect to the “no-DHA” metabolomic profiles (Figure 6 and Table 4).

As in the lipidome, no differences in the metabolome were detected after supplementation with PCA for 6 or 24 h (Table 4 and Table S6).

Very small effects attributable to PRO supplementation were visible after 6 h, becoming more evident after 24 h (Table 4 and Table S7). The PCA-CA discrimination accuracy for the comparison between “PRO” group (PRO, DHA + PRO) and “no-PRO” group (C, PCA, DHA, DHA + PCA) at time point 24 h was around 85%. Creatine phosphate levels were lower in the “PRO” group with respect to “no-PRO” group after 6 h (*p*-value = 0.027), while no differences were detected after 24 h. A decrement of uridine 5′-monophosphate (UMP) levels in “PRO” group was also monitored after 24 h.

## 3. Discussion

The main aim of this study was to provide a thorough assessment of the overall effect of the supplementation with DHA in human hepatocytes. The choice of DHA as the main bioactive to be studied was related to its large array of reported health benefits [22], and to its very broad range of proposed mechanisms of action. Two additional non-lipid bioactives were studied: PCA, the major metabolite of most anthocyanins [23], and PRO. PRO is produced in the colon through fermentation of dietary fibers. It is absorbed by the colonocytes, drains into the portal vein, and around 90% of PRO quantity is metabolized by the liver [24,25]. PCA and PRO were chosen as additional bioactives to be studied since they share with DHA a lipid-lowering effect [18,19,20]. The PRO and PCA lipid-lowering effects could be based on different mechanisms of action; therefore, their possible synergism with DHA deserves attention. Recently, the beneficial effects of DHA, PRO and PCA on fat metabolism and inflammatory phenotype have been evidenced in human white fat cells, and the synergistic effect of combination of DHA with PRO and PCA has been highlighted [21].

As a first step, the cell lipidome was screened using a fast, untargeted NMR approach. This allowed us to achieve perfect discrimination between cells receiving DHA (alone or in combination with PRO or PCA) and other cells (not supplemented or supplemented with PRO or PCA alone). Although a large body of literature supports the influence of DHA on cell lipidome, such a striking separation reinforces the idea of a global rearrangement of the lipid environment induced by DHA.

NMR spectra were also analyzed using univariate analysis. The two kinds of analyses (univariate and multivariate) provided quite consistent results, although not completely overlapping. The incomplete overlapping is simply the consequence of the different natures of the two approaches, since in the multivariate analysis correlations among metabolites are more important than absolute intensities of a specific metabolite [26]. It is worth noting that only minor deviations between the two approaches were evidenced in this study.

Notably, GC and fluorimetric analyses, used as complementary methods to validate NMR results, confirmed the strong NMR discrimination.

The increased DHA concentration and neutral lipid accumulation after DHA supplementation, particularly evident after 24 h, point to an extensive rearrangement of the lipid environment occurring in the cell. It is generally accepted that supplemented DHA is mainly incorporated into cell membrane PL, and this is known to have fundamental effects on membrane function [27]. The poor affinity of cholesterol for LC-PUFA promotes the formation of highly disordered membrane (non-raft) domains [28], which coexist with highly ordered lipid rafts, that are enriched in sphingolipids and cholesterol. The decreased level of phosphocholine, an intermediate in the synthesis of phosphatidylcholine, observed in the NMR spectra of “DHA” group, could somehow reflect this extensive remodeling of the cell membrane induced by DHA supplementation. A decrease in phosphatidylcholine levels to obtain similar choline-containing lipid levels between the rafts and the surrounding plasma membrane has been reported [8]. Phosphocholine is also one of the binding targets of C-reactive protein (CRP), which is involved in the clearance of apoptotic and necrotic cells, and is produced by the liver in response to inflammation [29]. Further investigations are needed to elucidate whether the observed reduced level of phosphocholine has a role in the mechanism of DHA anti-inflammatory effects.

n-3 LC-PUFA displace cholesterol from rafts, and internalize it into the cell. In agreement, the concentration of LD, lipid-rich cellular organelles that regulate the storage and hydrolysis of neutral lipids and serve as a reservoir for cholesterol and acyl-glycerols for membrane formation and maintenance [30], increased in all DHA supplemented cells. The increased content of neutral lipids in the “DHA”-group was confirmed by the higher TG signals in the NMR spectra. The observed increased storage of fat into LD in cells supplemented with DHA is in disagreement with other studies, indicating a suppression of LD formation [31], or no effect [32]. These discrepancies could be due not only to the different DHA concentrations used in the cited studies (100 and 10 µM, respectively), but mainly to the different cell types (3T3-L1 adipocytes and human SGBS pre-adipocyte, respectively). Effects of DHA supplementation in liver cells could be different than in other cell types, due to the crucial and complex role of liver cells in TG and cholesterol metabolism. Hepatocytes not only synthesize both TG and cholesterol, but they also receive the dietary TG and cholesterol contained in the chylomicron remnants. In cultured human L02 liver cells, TG levels significantly increased after supplementation of 3.2 or 12.8 µg/mL DHA [33]. The effective conversion of DHA into TG could represent a protective mechanism towards too high a DHA concentration in cell membrane PL.

In the present study, both the fluorimetric assay and NMR analysis evidenced a decrease of cholesterol concentration in DHA-supplemented hepatocytes. Although Nagao et al. [34] demonstrated that DHA supplementation does not affect cholesterol synthesis in HepG2 cells, gas chromatography-mass spectrometry-targeted metabolomic profiling has evidenced that DHA directly inhibits the activity of 3-hydroxy-3-methylglutaryl-coenzyme A (HMG-CoA) reductase, the first rate-limiting step of cholesterol biosynthesis [35]. Liver cells not only synthesize cholesterol, but also regulate its efflux via different metabolic pathways (i.e., very low-density lipoprotein (VLDL) secretion, bile acid synthesis). In cultured rat liver cells, VLDL biogenesis is reduced by DHA through presecretory proteolysis of apolipoprotein B (ApoB) [36], while bile acid-dependent cholesterol secretion is increased by fish oil [37]. Further studies are needed to completely elucidate the mechanisms involved in the regulation of cholesterol metabolism by DHA.

Using an untargeted approach based on multivariate statistics and the whole NMR metabolic fingerprint, we have shown that not only the lipidome, but also the metabolic profile radically changes after supplementation with DHA, independent of co-supplementation of PRO or PCA. As in the case of the fatty acid profile, the effects were stronger after 24 h. DHA administration increased the cellular levels of GSH and *O*-acetylcholine, and decreased the levels of aspartate, threonine and valine.

GSH is a key antioxidant, capable of preventing damage to important cellular components caused by reactive oxygen species [38]. GSH level is considered an important determinant of cellular redox status [39]. Our data on the increase in GSH levels after DHA supplementation are in agreement with Di Nunzio et al. [40,41], and confirm that DHA, besides being a highly oxidizable molecule [42], possesses antioxidant characteristics, including the induction of GSH.

Our data confirm that DHA markedly influences amino acid profiles, as observed in plasma, brain, liver and skeletal muscle of young pigs [43]. Since threonine and valine are essential amino acids, the decrease in their intracellular concentration in DHA group cannot be attributed to a decreased synthesis. It is therefore conceivable that an increased degradation of these two amino acids occurs. DHA could burst the citric acid cycle through the conversion of threonine and valine into succinyl-CoA [44], thereby enhancing the catabolism of sugar and fats and the production of energy. This could be an additional explanation for the beneficial effect of DHA in insulin resistance and diabetes [45], metabolic syndrome [46], and non-alcoholic fatty liver disease [47]. Aspartate is a non-essential amino acid in mammals, as it is produced from oxaloacetate by aspartate aminotransferase (AST). Further studies are needed to understand the biological and functional significance of the aspartate decrease, as well as of the higher concentration of acetylcholine observed in all cells supplemented with DHA. In nervous cells, the increase in acetylcholine concentration is considered one of the mechanisms at the basis of the claimed beneficial effect of DHA in cognitive development and memory [48], and in neurological disorders in childhood and adulthood [49]. At present, the functional effect of the observed increase of acetylcholine in liver cells is unknown.

## 4. Materials and Methods

### 4.1. Materials

Dulbecco’s modified Eagle’s medium (DMEM) and Dulbecco’s phosphate-buffered saline (DPBS) were purchased from Lonza (Basel, Switzerland). All other chemicals, reagents, and solvents were purchased from Sigma-Aldrich Co. (St. Louis, MO, USA), unless otherwise stated. All aqueous solutions were prepared using ultrapure water (Milli-Q; Millipore, Bedford, CT, USA).

### 4.2. Cell Culture and Supplementation

HepG2 human hepatoma cells were kept at 37 °C, 95% air, 5% CO_2_ in DMEM supplemented with 10% (*v/v*) fetal bovine serum (FBS), 100 U/mL penicillin and 100 mg/mL streptomycin. Once a week, cells were split 1:20 into a new 75 cm^2^ flask, and medium was refreshed. Cells were seeded at a density of 0.6 × 10^6^ cells (for fatty acid and lipid quantification) or at 1.0 × 10^6^ cells (for NMR experiments). After 24 h, at 75%–80% confluence, cells were divided randomly into six groups: (i) not supplemented (NS); (ii) supplemented with 70 µM PRO; (iii) supplemented with 20 µM PCA; (iv) supplemented with 50 µM DHA; (v) supplemented with 50 µM DHA + 70 µM PRO; (vi) supplemented with 50 µM DHA + 20 µM PCA.

DHA was dissolved in 100% ethanol, and complexed to bovine serum albumin (BSA); fatty acid–BSA complexes were prepared fresh each time at a final BSA concentration of 0.5% in serum-free DMEM. PCA was dissolved in dimethyl sulfoxide (DMSO) acidified with HCl (at pH 2), while PRO was dissolved in water. The final concentration of ethanol and DMSO was kept below 0.1% in serum-free DMEM. Control cells received corresponding amounts of BSA, ethanol and DMSO. DHA, PRO and PCA concentrations used in the study were evidenced to be non-cytotoxic in preliminary experiments.

### 4.3. Lipid Extraction and Fatty Acid Composition Analysis

After 6 or 24 h of supplementation, the medium was removed; cells were washed twice with warm DPBS, incubated with trypsin-EDTA for 2 min to remove adherent cells, and suspended in DMEM supplemented with 10% (*v/v*) FBS. Cell number in the suspension was determined using a TC20™ Automated Cell Counter (Bio-Rad Laboratories, Hercules, CA, USA).

Cell total lipids were extracted according to Folch et al. [50], and washings were analyzed by GC to ensure that supplemented FA had been completely removed and did not interfere with subsequent analyses. Fatty acids in the total lipid fraction were methylated according to Stoffel et al. [51]. Before methylation, pentadecanoic acid was added as internal standard.

Fatty acid (as methyl esters) profile was determined by GC (Clarus 500; PerkinElmer, Shelton, CT, USA) using a capillary column (SP2340, 0.2 µm film thickness) with a programmed temperature gradient (60–240 °C, 4 °C/min), as previously reported [4]. The gas chromatographic peaks were identified based on their retention time ratios relative to methyl stearate and predetermined by use of authentic samples. Gas chromatographic traces and quantitative evaluations were obtained using a Total-Chrom Navigator (version 6.2.1) (PerkinElmer, Shelton, CT, USA).

### 4.4. Evaluation of Lipid Accumulation and Cholesterol Concentration

The effect of the bioactives on lipid accumulation was evaluated by staining lipid droplets with Oil Red O [21], which allows detecting neutral lipids and lipid droplet morphology. Briefly, cells were fixed with 4% formalin in DPBS for two hours, washed with water, rinsed with isopropanol 60% and stained with Oil Red O for 30 min at room temperature. After washing with distilled water three times, the lipid droplets were quantified by dissolving Oil Red O in isopropanol 100% and measuring the optical density at 500 nm.

To evaluate the cholesterol content, the cellular lipids were extracted as previously described [52]. Total cholesterol and free cholesterol were quantified using the Amplex Red Cholesterol Assay Kit (Life Technologies Inc., Camarillo, CA, USA) according to the manufacturer’s instructions with the Infinite M200 microplate reader (Tecan; Salzburg, Austria). Samples were run with and without esterase to allow quantification of total cholesterol and free cholesterol; cholesterol ester concentration was estimated by subtracting the free cholesterol from total cholesterol. The results were expressed as micromolar concentrations of cholesterol per well.

### 4.5. Nuclear Magnetic Resonance (NMR) Sample Preparation

Cells were scraped off; the cell pellet was washed with ice-cold M9 buffer to remove traces of growth medium, and dissolved with 400 µL ice-cold DPBS buffer. Cells were lysed by sonication for 150 s by repeating the pulse 2 s on and 3 s off, then extracted using 800 µL of methanol-chloroform 1:1 (*v/v*) pre-cold at −80 °C. After mixing, vortex cells were incubated on ice for 30 min, and centrifuged at 14,000× *g* for 20 min at 4 °C. The resulting two phases were divided carefully, and the extraction procedure was repeated twice for each sample.

To prepare the water-soluble extracts, for each sample, the whole volume was evaporated, and the dried water-soluble cell extract was dissolved in 750 µL of phosphate sodium buffer (70 mM Na_2_HPO_4_; 20% (*v/v*) ^2^H_2_O; 0.025% (*v/v*) NaN_3_; 0.8% (*w/v*) sodium trimethylsilyl [2,2,3,3-^2^H_4_]propionate (TMSP) pH 7.4). A total of 600 µL of the extract were then transferred into 5 mm NMR tubes (Bruker BioSpin srl; Rheinstetten, Germany) for the analysis.

To prepare the lipid extract, for each sample, the whole volume was evaporated and the dried lipid cell extract was dissolved in 750 µL of CDCl_3_, and 600 µL were transferred into 5 mm NMR tubes (Bruker BioSpin srl; Rheinstetten, Germany) for the analysis.

### 4.6. NMR Sample Acquisition

In order to study the possible metabolomic changes induced by the cellular supplementation with DHA, PCA and PRO, alone or in combination, ^1^H-NMR spectra were acquired on HepG2 cell water-soluble and lipid extracts verifying their reproducibility (Figures S6 and S7). ^1^H-NMR spectra for all samples were acquired using a Bruker 600 MHz spectrometer (Bruker BioSpin srl; Rheinstetten, Germany) operating at 600.13 MHz proton Larmor frequency and equipped with a 5 mm PATXI ^1^H-^13^C-^15^N and ^2^H-decoupling probe including a *z* axis gradient coil, an automatic tuning-matching (ATM) and an automatic and refrigerated sample changer (SampleJet, Bruker BioSpin srl; Rheinstetten, Germany). A BTO 2000 thermocouple served for temperature stabilization at the level of approximately 0.1 K at the sample. Before measurement, samples were kept for 5 min inside the NMR probe head, for temperature equilibration at 300 K. For each sample, a monodimensional ^1^H NMR spectra was acquired with water peak suppression and a standard NOESY (Nuclear Overhauser Effect Spectroscopy) pulse sequence using 32 scans; 98,304 data points; a spectral width of 18,028 Hz; an acquisition time of 2.7 s; a relaxation delay of 4 s; and a mixing time of 0.1 s [53].

### 4.7. NMR Spectra Processing and Spectral Analysis

Free induction decays were multiplied by an exponential function equivalent to a 0.3 Hz line-broadening factor before applying Fourier transform. Transformed spectra were automatically corrected for phase and baseline distortions and calibrated (reference signal of TMSP at δ 0.00 ppm for water-soluble extracts and chloroform singlet at 7.25 ppm for lipid extracts) using TopSpin 3.5 (Bruker BioSpin srl; Rheinstetten, Germany). Each spectrum of the water-soluble extracts, in the region 10.00–0.2 ppm, was segmented into 0.02 ppm chemical shift bins, and the corresponding spectral areas were integrated using the AMIX software (Bruker BioSpin srl; Rheinstetten, Germany). Binning is a means to reduce the number of total variables and to compensate for small shifts in the signals, making the analyses more robust and reproducible. The area of each bin was normalized to the total spectral area, calculated with exclusion of the water region (4.50–5.20 ppm). Each spectrum of the lipid extracts, in the region 6.5–0.2 ppm, was segmented into 0.02 ppm chemical shift bins, and the corresponding spectral areas were integrated using the AMIX software (Bruker BioSpin srl; Rheinstetten, Germany). The area of each bin was normalized to the total spectral area.

### 4.8. Statistical Analysis

Resulting data on and fatty acid composition, lipid accumulation and cholesterol concentration are given as mean ± standard deviation (SD). The statistical differences were determined by the one-way analysis of variance (ANOVA) followed by Dunnett’s test for comparison with NS cells, considering *p* < 0.05 as significant.

Various kinds of multivariate statistical techniques were applied on the obtained buckets using R 3.0.2 in house scripts. Univariate statistical analyses ware performed on selected assigned and integrated spectral signals using R 3.0.2 in house scripts. Principal component analysis (PCA) was used to obtain a preliminary overview of the data (visualization in a reduced space, clusters detection, screening for outliers); canonical analysis (CA) was used in combination with PCA to perform supervised data reduction and classification. Accuracy, specificity and sensitivity were estimated according to standard definitions. The global accuracy for classification was assessed by means of a leave-one-out cross-validation scheme. There were 23 metabolites and 15 lipid components, whose peaks in the spectra were well defined and resolved, analyzed in the water-soluble extracts and lipid extracts, respectively. Signal identification was performed using a library of NMR spectra of pure organic compounds, public databases (such as HMBD, Human Metabolic Database, and SDBS, Spectra Database for Organic Compounds) storing reference NMR spectra of metabolites, spiking NMR experiments and literature data [54,55]. The relative concentrations of the various metabolites and lipid groups in the different spectra were calculated by integrating the signal area [56]. The Wilcoxon test was used for the determination of the meaningful signals: a *p*-value of 0.05 was considered statistically significant. The effect size, using the Cliff’s delta (Cd) formulation [57], was also calculated to aid the identification of the meaningful signals giving an estimation of the magnitude of the separation between the different groups. The magnitude is assessed using the thresholds provided in Romano et al. [58], i.e., |Cd| < 0.147 “negligible”, |Cd| < 0.33 “small”, |Cd| < 0.474 “medium”, otherwise “large”.

### 4.9. Data Deposition

The NMR data have been uploaded in the MetaboLights database (www.ebi.ac.uk/metabolights) [59]. They will be accessible (accession number MTBLS419) once the curation phase due to data processing is concluded.

## 5. Conclusions

Docosahexaenoic acid (DHA) plays important and different roles in regulating various aspects of cellular life, affecting the concentration of lipids, fatty acids, amino acids, and metabolites. In cultured hepatocytes, a “DHA signature”—already present after 6 h and even stronger after 24 h supplementation—clearly indicated the strong perturbation of the cell environment after DHA supplementation. On the contrary, supplementation with the other tested metabolites did not cause an extensive perturbation of the cell environment, not allowing discrimination between supplemented and non-supplemented cells. The “DHA signature” allowed for the discrimination of cells supplemented with the fatty acid, regardless of the co-supplementation with the other bioactives. Interestingly, the “DHA signature” was not only related to the lipidome, but also to the metabolome. Metabolites are the final products of cellular activities, and their levels change according to environmental factors and external stimuli. Therefore, metabolomics represents a powerful tool to understand—at the molecular level—the effects of bioactive supplementation [60,61].

The results reported here indicate that nuclear magnetic resonance (NMR), subsequently confirmed by other techniques, represents a fundamental approach for studying the effect of the supplementation of bioactives, particularly in the case of molecules with a broad spectrum of mechanisms of action. In fact, it allows both the characterization of the lipophilic fraction and the metabolic profiling of the water-soluble fraction (cytoplasmic metabolome) in a fast and completely untargeted approach. The additional information thus far accessed by metabolomics allows the follow-up of cellular metabolic perturbations consequent to cascade pleiotropic mechanisms activated by the cells to adapt their biological processes to a different environment, including changes in the lipidomic profile affecting the membrane structure.

The evidence of the perturbation of cell lipidome and metabolome does not represent a mechanism of action; rather, the main metabolites involved in this perturbation indicate possible mechanisms of action that need to be investigated in other studies. To shed light on these complex mechanisms, further work is still needed. The use of complementary approaches, such as targeted mass spectrometry, could be helpful to obtain better insight into the metabolic pathways involved in the cellular response to different bioactives, and in the mechanisms underlying the bioactives’ health effects.

## Figures and Tables

**Table 1:** List of lipid groups whose signals were assigned and integrated in the nuclear magnetic resonance (NMR) spectra. For each comparison, the corresponding *p*-value of the Wilcoxon test and the Cliff’s delta (Cd) effect size are reported (* *p* < 0.05; ** *p* < 0.01; *** *p* < 0.001, n = negligible, s = small, m = medium, l = large). The signals whose intensities were significantly higher or lower in “DHA”, “PCA” and “PRO” groups are marked with ↑ or ↓, respectively.

Metabolites	Moieties Assignment	“DHA” vs. “No-DHA”		“PRO” vs. “No-PRO”		“PCA” vs. “No-PCA”	
		*p*-Value 6 h	*p*-Value 24 h	*p*-Value 6 h	*p*-Value 24 h	*p*-Value 6 h	*p*-Value 24 h
Total cholesterol	C18-**H_3_**	0.308 (s)	7.2 × 10^−6^ ***↓ (l)	0.895 (n)	0.566 (n)	0.537 (s)	0.825 (n)
FA, ω-CH_3_	FA chain C**H_3_**(CH_2_)*_n_*	0.00268 **↓ (l)	0.780 (n)	0.453 (s)	0.964 (n)	0.311 (s)	0.894 (n)
ω-3, (e.g., DHA * + EPA + linoleic)	ω-3 C**H_3_**–CH_2_-C=C	2.9 × 10^−6^ ***↑ (l)	2.9 × 10^−6^ ***↑ (l)	0.757 (n)	0.860 (n)	0.724 (n)	0.453 (s)
Free cholesterol	C19–**H_3_**	0.755 (n)	9.5 × 10^−6^ ***↓ (l)	0.628 (n)	0.825 (n)	0.233 (s)	0.537 (n)
Esterified cholesterol	C19–**H_3_**	0.070 (m)	0.119 (s)	0.691 (n)	0.453 (s)	0.597 (n)	0.537 (n)
FA (total fatty acyl chains)	FA chain –(C**H_2_**)*_n_*–	0.015 *↓ (l)	0.0235 *↓ (l)	0.724 (n)	0.537 (n)	0.310 (s)	0.272 (s)
FA, βH_2_	βH_2_ *R*–C**H_2_**–CH_2_–CO–OR	0.467 (s)	0.0034 **↑ (l)	0.402 (s)	0.929 (n)	1 (n)	0.158 (s)
FA	–CH=CH–C**H_2_**–	0.205 (s)	0.00032 ***↓ (l)	0.791 (n)	0.354 (s)	0.354 (s)	1 (n)
FA, αH_2_	αH_2_ –C**H_2_**–CO–OR	0.467 (s)	0.0057 **↑ (l)	0.354 (s)	0.172 (s)	0.825 (n)	0.0275 *↓ (l)
FA (e.g., DHA *)	α**H_2_** and β**H_2_** –CH=CH–C**H_2_**–C**H_2_**–CO–OR	2.9 × 10^−6^ ***↑ (l)	2.9 × 10^−6^ ***↑ (l)	0.566 (n)	0.791 (n)	0.453 (s)	0.480 (s)
FA (e.g., linoleic)	–CH=CH–C**H_2_**–(CH=CH–CH_2_–)*_n_*, *n* = 1	0.0396 *↑ (m)	0.0038 **↓ (l)	0.791 (n)	0.481 (s)	0.402 (s)	0.757 (n)
FA, PUFA (e.g., DHA *)	–CH=CH–C**H_2_**–(CH=CH–CH_2_–)*_n_*, *n* > 1	2.9 × 10^−6^ ***↑ (l)	2.9 × 10^−6^ ***↑ (l)	0.965 (n)	0.825 (n)	0.480 (s)	0.929 (n)
Phosphatidylcholine	CH_2_–N–(C**H_3_**)_3_	0.852 (n)	0.693 (n)	0.758 (n)	0.480 (s)	0.217 (s)	0.627 (n)
Triglycerides	glycerol (C1-**H^u^**) and (C3-**H^u^**)	0.164 (s)	4.4 × 10^−6^ ***↑ (l)	0.659 (n)	0.427 (s)	0.965 (n)	0.427 (s)
FA, MUFA and PUFA	–C**H**=C**H**–	2.9 × 10^−6^ ***↑ (l)	2.9 × 10^−6^ ***↑ (l)	1 (n)	0.724 (n)	0.481 (s)	0.895 (n)

DHA: docosahexaenoic acid; FA: fatty acids; MUFA: monounsaturated fatty acids; PCA: protocatechuic acid; PRO: propionic acid; PUFA: polyunsaturated fatty acids.

**Table 2:** Fatty acid content of not supplemented (NS) and supplemented cells after 6 h supplementation. Data were obtained by gas chromatography (GC) analysis, and are expressed as µg FA/10^6^ cells. Data in each group are means ± standard deviation (SD) of five samples derived from three independent experiments. Statistical analysis was conducted with the one-way ANOVA (22:6n-3 *p* < 0.001; UI *p* < 0.001; n-3/n-6 *p* < 0.001; ΣPUFA *p* < 0.001) using Dunnett’s post-test to compare NS and supplemented cells (** *p* < 0.01; *** *p* < 0.001).

FA	NS	70 µM PRO	20 µM PCA	50 µM DHA	50 µM DHA + 70 µM PRO	50 µM DHA + 20 µM PCA
3:0	0.06 ± 0.07	0.13 ± 0.09	0.03 ± 0.03	0.09 ± 0.10	0.04 ± 0.07	0.03 ± 0.04
14:0	1.30 ± 0.62	1.07 ± 0.64	1.11 ± 0.53	1.91 ± 1.23	1.63 ± 0.13	1.61 ± 0.09
16:0	22.37 ± 7.28	21.71 ± 4.67	21.08 ± 3.40	24.71 ± 8.04	26.6 ± 4.85	23.65 ± 1.32
16:1n-7	1.63 ± 0.28	1.85 ± 0.76	1.93 ± 0.39	2.04 ± 0.62	2.21 ± 0.59	2.21 ± 0.15
18:0	19.95 ± 7.96	18.9 ± 3.58	17.70 ± 2.65	19.90 ± 6.87	21.87 ± 3.89	19.19 ± 2.41
18:1n-9	5.85 ± 1.13	7.30 ± 2.47	6.76 ± 1.76	7.74 ± 2.59	7.48 ± 2.17	6.81 ± 1.00
18:1n-7	6.84 ± 1.09	7.60 ± 2.28	7.56 ± 1.64	6.30 ± 1.46	7.65 ± 2.36	6.84 ± 0.84
18:2n-6	0.26 ± 0.11	0.27 ± 0.08	0.26 ± 0.06	0.36 ± 0.28	0.33 ± 0.05	0.28 ± 0.05
18:3n-3	0.37 ± 0.13	0.32 ± 0.13	0.31 ± 0.15	0.53 ± 0.25	0.44 ± 0.11	0.37 ± 0.05
20:4n-6	6.19 ± 1.36	7.43 ± 1.96	7.52 ± 0.89	6.77 ± 1.37	8.93 ± 1.47	7.94 ± 1.32
20:5n-3	0.25 ± 0.06	0.24 ± 0.05	0.29 ± 0.21	0.32 ± 0.16	0.53 ± 0.52	0.24 ± 0.02
22:6n-3	0.92 ± 0.46	0.61 ± 0.37	0.67 ± 0.23	6.16 ± 1.07 ***	6.98 ± 2.48 ***	6.31 ± 0.80 ***
UI	45.87 ± 9.08	50.97 ± 13.88	51.32 ± 7.67	81.99 ± 16.46 ***	97.38 ± 22.80 ***	86.17 ± 4.30 ***
n-3/n-6	0.24 ± 0.09	0.15 ± 0.02	0.16 ± 0.06	1.00 ± 0.15 ***	0.87 ± 0.28 ***	0.87 ± 0.25 ***
ΣSFA	43.68 ± 15.7	41.81 ± 8.68	39.92 ± 6.29	46.61 ± 16.08	50.13 ± 8.88	44.48 ± 3.61
ΣMUFA	14.32 ± 1.06	16.75 ± 5.45	16.25 ± 3.70	16.09 ± 4.59	17.34 ± 5.09	15.87 ± 1.70
ΣPUFA	8.00 ± 1.91	8.87 ± 2.22	9.05 ± 1.07	14.14 ± 2.87 **	17.21 ± 3.41 ***	15.15 ± 0.88 ***
Total FA	66.00 ± 16.66	67.42 ± 15.73	65.21 ± 10.15	76.83 ± 23.03	84.69 ± 17.18	75.49 ± 4.13

DHA: docosahexaenoic acid; FA: fatty acids; MUFA: monounsaturated fatty acids; NS: not supplemented; PCA: protocatechuic acid; PRO: propionic acid; PUFA: polyunsaturated fatty acids; SFA: saturated fatty acids; UI: unsaturation index.

**Table 3:** Fatty acid content of not supplemented (NS) and supplemented cells after 24 h supplementation. Data were obtained by GC analysis, and are expressed as µg FA/10^6^ cells. Data in each group are means ± SD of five samples derived from three independent experiments. Statistical analysis was performed by one-way ANOVA (14:0 *p* < 0.01; 16:0 *p* < 0.001; 18:0 *p* < 0.01; 18:2n-6 *p* < 0.001; 20:4n-6 *p* < 0.05; 22:6n-3 *p* < 0.001; UI *p* < 0.001; n-3/n-6 *p* < 0.001; ΣSFA *p* < 0.001; ΣPUFA *p* < 0.001; fatty acid content *p* < 0.001) using Dunnett’s post-test to compare NS and supplemented cells (* *p* < 0.05; ** *p* < 0.01; *** *p* < 0.001).

FA	NS	70 µM PRO	20 µM PCA	50 µM DHA	50 µM DHA + 70 µM PRO	50 µM DHA + 20 µM PCA
3:0	0.35 ± 0.10	0.21 ± 0.05	0.41 ± 0.51	0.56 ± 0.55	0.61 ± 0.79	0.38 ± 0.21
14:0	1.65 ± 0.28	1.12 ± 0.17	1.16 ± 0.12	1.75 ± 0.64	1.94 ± 0.37	2.07 ± 0.39
16:0	15.82 ± 2.64	9.45 ± 0.81 **	9.30 ± 1.70 **	14.61 ± 2.86	14.13 ± 2.39	16.17 ± 3.81
16:1n-7	2.40 ± 0.16	1.83 ± 0.11	1.77 ± 0.43	1.98 ± 0.28	2.14 ± 0.39	2.29 ± 0.45
18:0	14.54 ± 4.45	6.06 ± 2.05 **	5.46 ± 0.92 ***	11.21 ± 3.57	8.85 ± 1.68 *	10.00 ± 3.19
18:1n-9	8.96 ± 0.74	6.98 ± 0.81	6.68 ± 1.55	7.34 ± 0.53	7.81 ± 1.40	8.79 ± 1.99
18:1n-7	5.68 ± 0.40	4.46 ± 0.66	4.33 ± 0.99	3.93 ± 0.23 *	4.22 ± 0.65	4.63 ± 0.85
18:2n-6	0.43 ± 0.07	0.31 ± 0.04 *	0.30 ± 0.07 *	0.21 ± 0.01 ***	0.22 ± 0.04 ***	0.23 ± 0.05 ***
18:3n-3	0.45 ± 0.05	0.35 ± 0.07	0.34 ± 0.09	0.33 ± 0.02	0.34 ± 0.07	0.40 ± 0.09
20:4n-6	0.84 ± 0.17	0.69 ± 0.20	0.67 ± 0.13	0.70 ± 0.19	0.93 ± 0.18	1.09 ± 0.05
20:5n-3	0.35 ± 0.31	0.26 ± 0.30	0.24 ± 0.32	0.18 ± 0.21	0.12 ± 0.21	0.38 ± 0.31
22:6n-3	1.36 ± 0.33	1.01 ± 0.45	0.92 ± 0.42	8.28 ± 0.85 ***	8.92 ± 1.34 ***	10.45 ± 2.02 ***
UI	30.13 ± 4.21	23.20 ± 5.57	22.00 ± 7.26	66.11 ± 5.39 ***	71.37 ± 10.36 ***	84.01 ± 16.55 ***
n-3/n-6	1.73 ± 0.65	1.68 ± 0.98	1.49 ± 0.59	10.20 ± 3.57 ***	8.24 ± 1.04 ***	8.49 ± 1.63 ***
ΣSFA	32.35 ± 7.26	16.85 ± 2.70 **	16.32 ± 2.99 **	28.13 ± 6.78	25.53 ± 4.21	28.62 ± 6.99
ΣMUFA	17.05 ± 1.29	13.27 ± 1.40	12.78 ± 2.85	13.26 ± 0.91	14.17 ± 2.44	15.72 ± 3.29
ΣPUFA	3.43 ± 0.59	2.61 ± 0.78	2.46 ± 0.97	9.71 ± 0.86 ***	10.54 ± 1.47 ***	12.54 ± 2.4 ***
Total FA	52.83 ± 7.20	32.73 ± 4.84 **	31.56 ± 6.67 **	51.09 ± 6.78	50.24 ± 7.78	56.88 ± 12.36

DHA: docosahexaenoic acid; FA: fatty acids; MUFA: monounsaturated fatty acids; NS: not supplemented; PCA: protocatechuic acid; PRO: propionic acid; PUFA: polyunsaturated fatty acids; SFA: saturated fatty acids; UI: unsaturation index.

**Table 4:** List of metabolites whose signals were assigned and integrated in the nuclear magnetic resonance (NMR) spectra. For each metabolite, the integration range is reported. For each comparison, the corresponding *p*-value of the Wilcoxon test and the Cliff’s delta (Cd) effect size are reported (* *p* < 0.05; ** *p* < 0.01; *** *p* < 0.001, n = negligible, s = small, m = medium, l = large).

Metabolites (ppm)	“DHA” vs. “No-DHA”				“PRO” vs. “No-PRO”				“PCA” vs. “No-PCA”			
	6 h		24 h		6 h		24 h		6 h		24 h	
	*p*-Value	Cd	*p*-Value	Cd	*p*-Value	Cd	*p*-Value	Cd	*p*-Value	Cd	*p*-Value	Cd
Isoleucine (0.945–0.938)	0.917	n	1	n	0.112	s	0.456	s	1	n	0.380	s
Valine (1.003–0.981)	0.662	n	0.036 *	m	0.567	n	0.312	s	0.691	n	0.757	n
Lactate/threonine (1.346–1.320)	0.950	n	0.025 *	l	0.691	n	0.062	m	0.047	m	0.302	s
Alanine (1.496–1.473)	0.519	n	0.072	s	0.112	m	0.594	s	0.965	n	0.718	n
Acetate (1.929–1.917)	0.755	n	0.697	n	0.628	n	0.099	m	0.427	s	0.57	s
Glutamate (2.081–2.034)	0.884	n	0.961	n	0.252	s	0.632	n	0.758	n	0.099	m
Glutathione (2.584–2.534)	0.177	s	0.006 **	l	0.691	n	0.632	n	0.597	n	0.439	s
Creatine (3.044–3.038)	0.390	s	0.098	m	0.427	s	0.424	s	0.354	s	0.38	s
Phosphocreatine (3.052–3.044)	0.152	s	0.733	n	0.027*	l	0.221	s	1	n	0.718	n
Glutamine (2.168–2.109)	0.662	n	0.592	n	0.332	s	0.338	s	0.234	s	0.959	n
*O*-phosphocholine (3.229–3.215)	0.036 *	m	0.661	n	0.086	m	0.958	n	0.354	s	0.164	s
Methanol (3.373–3.358)	0.755	n	0.013 *	l	0.724	n	0.79	s	0.724	n	0.164	m
Succinate (2.410–2.403)	0.467	s	0.496	s	0.064	m	0.11	m	0.597	n	0.796	n
Formate (8.466–8.451)	0.983	n	0.206	s	0.965	n	0.632	n	1	n	0.439	s
Pantothenate (0.903–0.894)	0.603	n	0.206	s	0.724	n	0.749	n	0.186	s	0.439	s
Aspartate (2.808–2.793)	0.036 *	m	0.088	m	0.27	s	0.15	m	0.965	n	0.409	s
Glycine (3.573-3.561)	0.519	n	0.173	s	0.481	s	0.394	s	0.454	s	0.409	s
Threonine (4.265–4.256)	0.493	n	0.036 *	m	0.078	m	0.67	n	0.311	s	0.353	s
AMP/IMP (4.525–4.501)	0.575	n	0.527	n	0.217	s	0.338	n	0.481	s	0.235	s
UMP (8.121-8.098)	0.633	n	0.559	n	0.234	s	0.019 *	m	0.332	s	0.536	n
*O*-acetylcholine (3.236–3.229)	2 × 10^−5^ ***	l	0.017 *	l	0.826	s	0.043	m	0.930	n	0.796	n
Leucine (0.969–0.960)	0.547	n	0.381	s	0.332	s	0.523	n	0.965	n	0.256	s

AMP: adenosine monophosphate; DHA: docosahexaenoic acid; IMP: inosine monophosphate; PCA: protocatechuic acid; PRO: propionic acid; UMP: uridine 5′-monophosphate.

## Supplementary Materials

Supplementary materials can be found at www.mdpi.com/1422-0067/18/2/359/s1.

## Abbreviations

A	Actual classes
AMP	Adenosine monophosphate
ANOVA	Analysis of variance
ApoB	Apolipoprotein B
AST	Aspartate aminotransferase
ATM	Automatic tuning-matching
BSA	Bovine serum albumin
Cd	Cliff’s delta
CRP	C-reactive protein
DHA	Docosahexaenoic acid
DMEM	Dulbecco’s modified Eagle’s medium
DMSO	Dimethyl sulfoxide
DPBS	Dulbecco’s phosphate-buffered saline
DRD	Diet related diseases
EDTA	Ethylenediaminetetraacetic acid
EFSA	European Food Safety Authority
FA	Fatty acids
FBS	Fetal bovine serum
FDA	Food and Drug Administration
GC	Gas chromatography
GSH	Reduced glutathione
H NMR	Proton nuclear magnetic resonance
HDL	High-density lipoproteins
HMBD	Human metabolic database
HMG-CoA	3-Hydroxy-3-methylglutaryl-coenzyme A
IMP	Inosine monophosphate
l	Large
LC-MS/MS	Liquid chromatography-tandem mass spectrometry
LC-PUFA	Long-chain polyunsaturated fatty acids
LD	Lipid droplets
LDL	Low-density lipoproteins
m	Medium
MUFA	Monounsaturated fatty acids
NMR	Nuclear magnetic resonance
NOESY	Nuclear Overhauser effect spectroscopy
n	Negligible
NS	Not supplemented
P	Predicted classes
PCA	Protocatechuic acid
PL	Phospholipids
PCA-CA	Principal component analysis and canonical analysis
PRO	Propionic acid
PUFA	Polyunsaturated fatty acids
RCT	Randomized controlled trial
s	Small
SD	Standard deviation
SDBS	Spectra database for organic compounds
SFA	Saturated fatty acids
TG	Triglycerides
TMSP	Trimethylsilylpropanoic acid
UI	Unsaturation index
UMP	Uridine 5′-monophosphate
VLDL	Very low-density lipoproteins
