# Supplementary material for: Evidence of a DHA Signature in the Lipidome and Metabolome of Human Hepatocytes
Source: Int J Mol Sci. 2017 Feb 8;18(2):359. doi: 10.3390/ijms18020359 (PMC5343894; doi:10.3390/ijms18020359)
Supplement: Supplementary file 1 [file ijms-18-00359-s001.pdf]

Supplementary Materials: Evidence of a DHA Signature in the Lipidome and Metabolome of Human Hepatocytes

Veronica Ghini, Mattia Di Nunzio, Leonardo Tenori, Veronica Valli, Francesca Danesi, Francesco Capozzi, Claudio Luchinat and Alessandra Bordoni

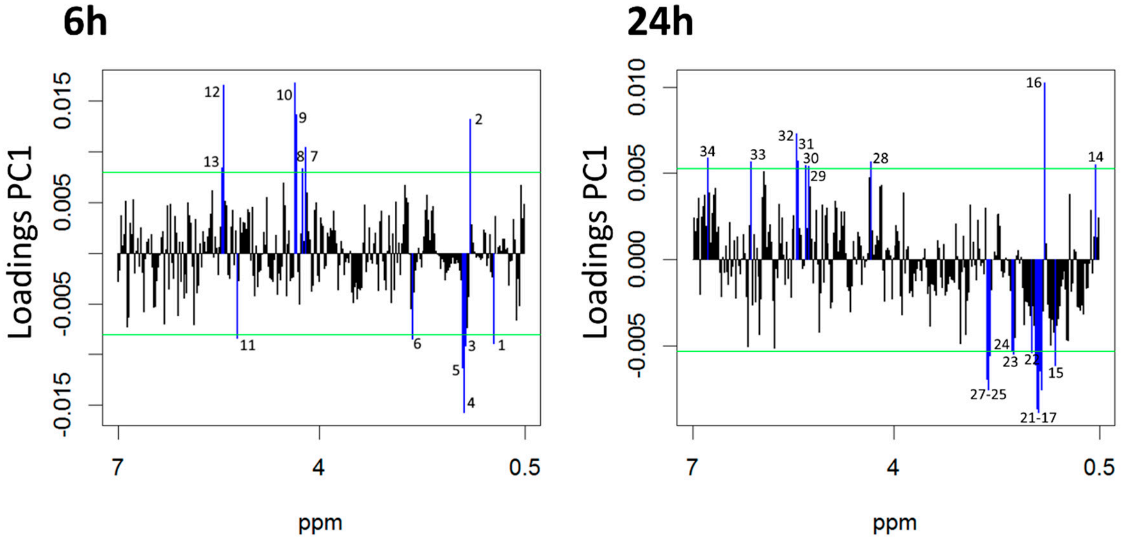

Figure S1. Lipidomic phenotyping by nuclear magnetic resonance (NMR) analysis of cells not supplemented and supplemented with docosahexaenoic acid (DHA), alone or in combination with protocatechuic acid (PCA) or propionic acid (PRO). PC1 loading plots of principal component analysis and canonical analysis (PCA-CA) discrimination of "DHA" group (DHA, DHA + PCA, DHA + PRO) and "no-DHA" group (NS, PRO, PCA). The significance threshold (green lines) was calculated considering "buckets" with a value beyond two standard deviations of their averages. (**Left panel**) 6 h data set; 1 TC (C21-$\mathbf{H}_3$); 2 unknown; 3–5 FA, (–(C$\mathbf{H}_2$)$_n$–); 6 FA (–CH=CH–C$\mathbf{H}_2$–); 7–10: TG (Glycerol (C1-$\mathbf{H}^d$) and (C3-$\mathbf{H}^d$)); 11–13: TG (Glycerol (C2-**H**)). (**Right panel**) 24 h data set; 14: unknown (–C$\mathbf{H}_3$); 15 TC (–C$\mathbf{H}_3$); 16 unknown; 17–21 FA, (–(C$\mathbf{H}_2$)$_n$–); 22–24: unknown; 25–27: FA (–CH=CH–C$\mathbf{H}_2$–); 28: TG (Glycerol (C1-$\mathbf{H}^d$) and (C3-$\mathbf{H}^d$)); 29–32: TG (Glycerol (C2-**H**)); 33–34: unknown. FA: fatty acids; TC: total cholesterol; TG: triglycerides.

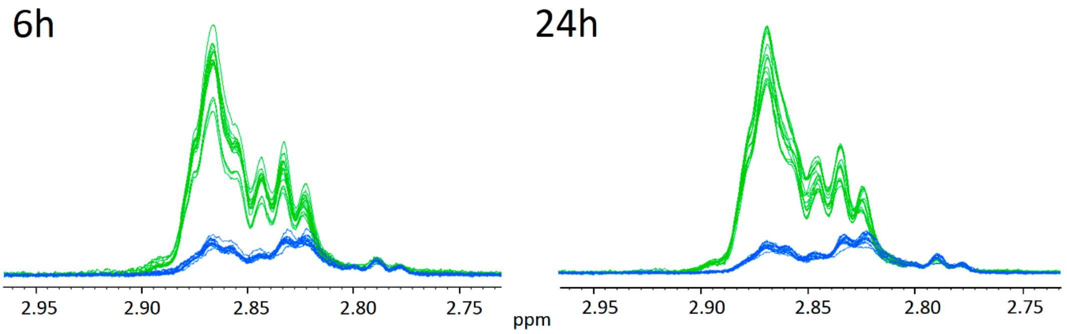

Figure S2. PUFA (e.g., DHA) NMR signal in "no-DHA" group (not supplemented (NS), protocatechuic acid (PCA), propionic acid (PRO)) and "DHA group" (docosahexaenoic acid (DHA), DHA + PCA, DHA + PRO). "no-DHA" group, blue traces; "DHA group", green traces. (**Left panel**) 6h data set, p-value = 3×10^{-6}; (**Right panel**) 24 h data set, p-value = 3×10^{-6}.

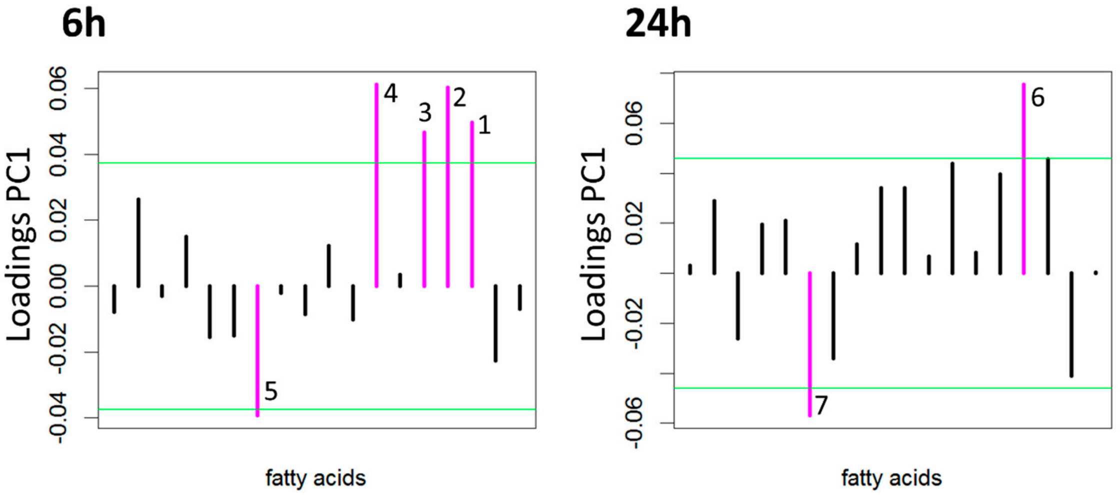

Figure S3. Fatty acid phenotyping by gas chromatography (GC) analysis of cells not supplemented (NS) and supplemented with docosahexaenoic acid (DHA), alone or in combination with protocatechuic acid (PCA) or propionic acid (PRO), PC1 loading plots of principal component analysis and canonical analysis (PCA-CA) discrimination of "DHA" group (DHA, DHA + PCA, DHA + PRO) and "no-DHA" group (NS, PRO, PCA). The significance threshold (green lines) was calculated considering "buckets" with a value beyond one standard deviation of their averages. (**Left panel**) 6 h data set; 1: C22:6 n-3 (DHA); 2: UI; 3: n-3/n-6; 4: ΣPUFA; 5: C18:1 n-7 (vaccenic acid); (**Right panel**) 24 h data set; 6: C18:1 n-9 (oleic acid); 7: n-3/n-6.

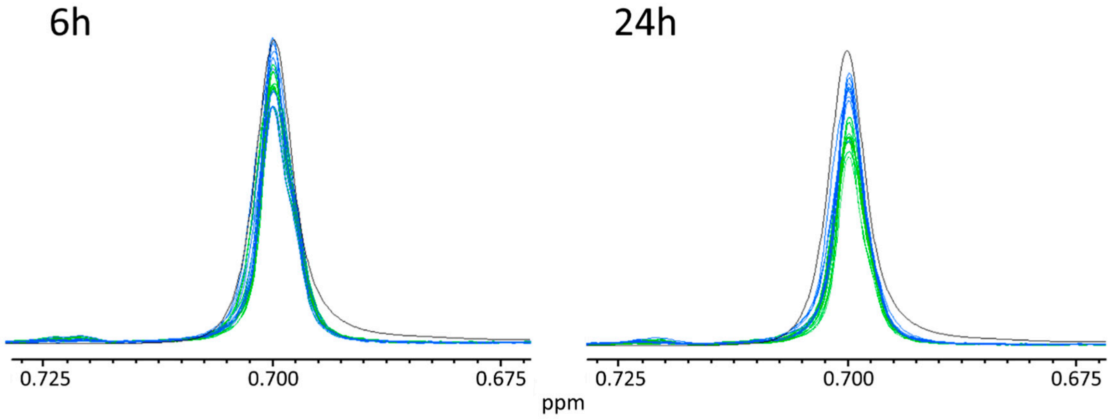

Figure S4. Cholesterol NMR signal in "no-DHA" group (not supplemented (NS), protocatechuic acid (PCA), propionic acid (PRO)) and "DHA group" (docosahexaenoic acid (DHA), DHA + PCA, DHA + PRO). "no-DHA" group, blue traces; "DHA group", green traces; cholesterol reference NMR spectrum, black trace. (**Left panel**) 6 h data set, p-value > 0.05; (**Right panel**) 24 h data set, p-value = 0.024.

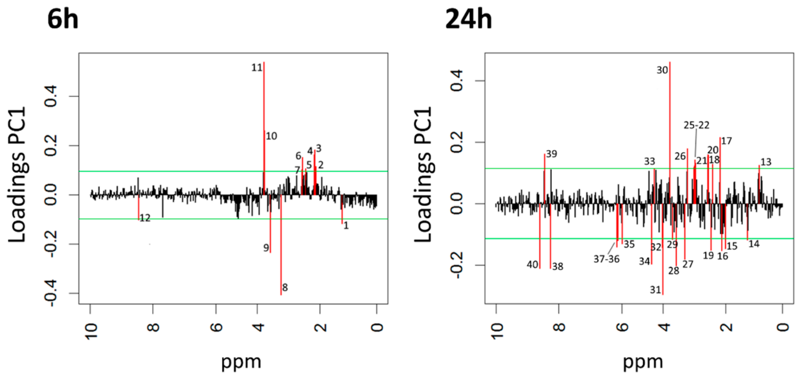

Figure S5. Metabolomic phenotyping by nuclear magnetic resonance (NMR) analysis of not supplemented (NS) and supplemented cells with docosahexaenoic acid (DHA), alone or in combination with protocatechuic acid (PCA) or propionic acid (PRO). PC1 loading plots of principal component analysis and canonical analysis (PCA-CA) discrimination of "DHA" group (DHA, DHA + PCA, DHA + PRO) and "no-DHA" group (NS, PRO, PCA). The significance threshold (green lines) was calculated considering "buckets" with a value beyond two standard deviations of their averages. (**Left panel**) 6 h data set; 1: unknown; 2–4: *O*-acetylcholine/glutathione/glutamate; 5: unknown; 6–7: glutathione; 8: *O*-acetylcholine; 9: *O*-phosphocholine/threonine/valine; 10–11: glutathione/glutamate; 12: formate. (**Right panel**) 24 h data set; 13–15: unknown; 16: *O*-acetylcholine/glutathione/glutamate; 17: glutathione/glutamate; 18: succinate; 19: unknown; 20–25: glutathione; 26: *O*-phosphocholine; 27: unknown; 28: *O*-phosphocholine/threonine/valine; 29: unknown; 30: glutathione/glutamate; 31–33: adenosine monophosphate (AMP)/inosine monophosphate (IMP); 34: unknown; 35: AMP/IMP; 36: uridine 5′-monophosphate (UMP); 37-38: AMP/IMP; 39: formate; 40: unknown.

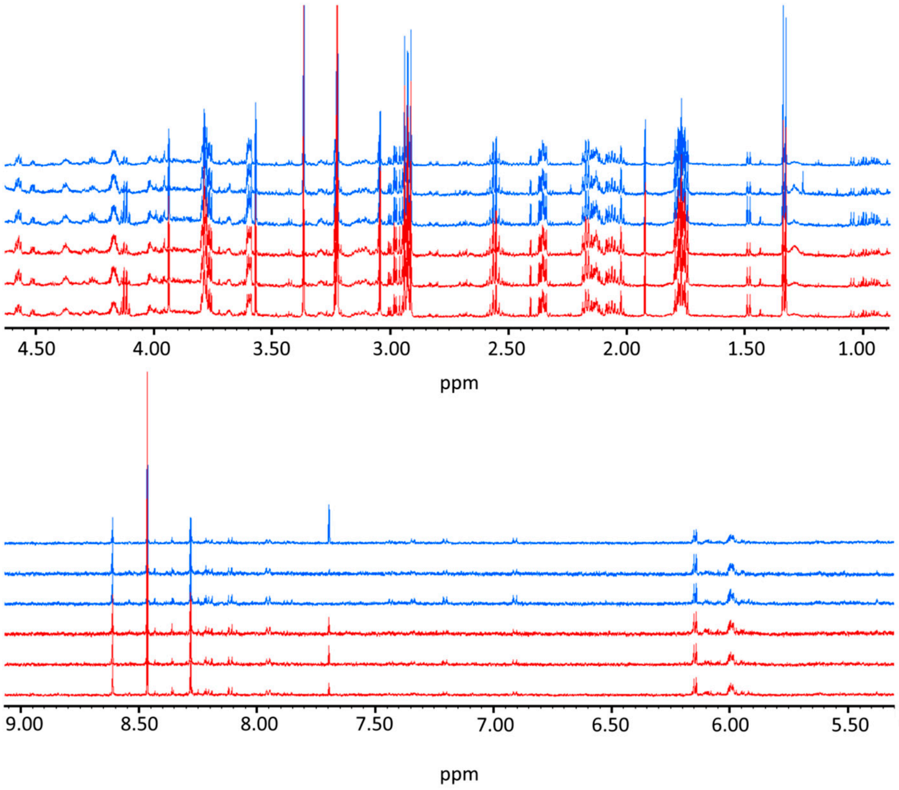

Figure S6. Upfield (1.00–4.50 ppm, **upper panel**) and downfield (5.50–9.00 ppm, **lower panel**) regions of the ^{1}H-NMR NOESY spectra of methanol extracts; for better peak visualization a 2× vertical expansion is used for the downfield region. Blue tracks: 3 spectra from control group; red tracks: 3 spectra from docosahexaenoic acid (DHA) group.

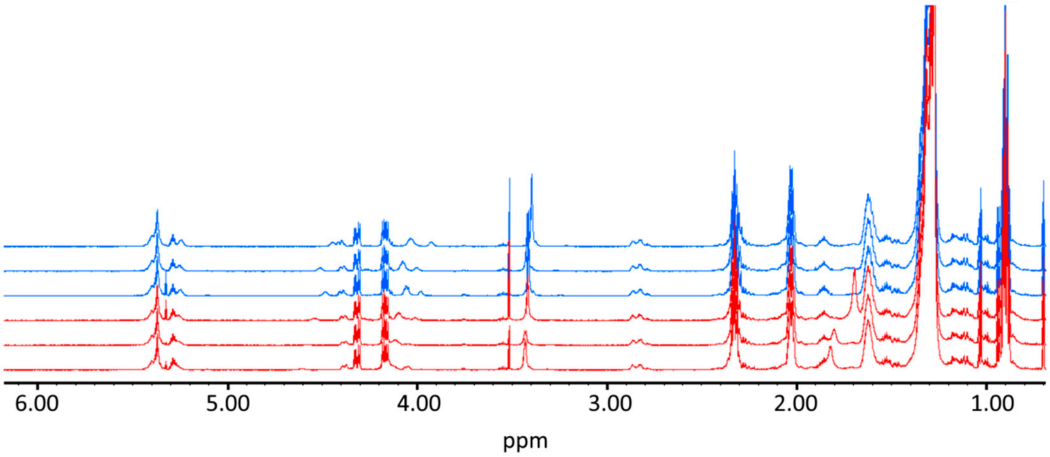

Figure S7. ^{1}H-NMR NOESY spectra of chloroform extracts. Blue tracks: 3 spectra from control group; red tracks: 3 spectra from docosahexaenoic acid (DHA) group.

Table S1. ^{1}H nuclear magnetic resonance (NMR) resonance assignment for signals identified in lipid-soluble extracts. * Spectral areas removed from the multivariate statistical analysis due to the presence of intense signals introduced by DHA; (u) signals used for the univariate statistical analysis.

Metabolite	δ ([1]H Chemical Shift) ppm	n° Protons	Moieties Assignment	Multiplicity
Total cholesterol (u)	0.70	3	C18-$\mathbf{H_3}$	s
Total cholesterol	0.86	3 (+3)	C26–C27–$\mathbf{H_3}$	2× d
FA, *ω*-CH_3 (u)	0.88	3	FA chain $\mathbf{CH_3}(CH_2)_n$	t (6.9)
Total cholesterol	0.92	3	C21-$\mathbf{H_3}$	d (6.6)
ω-3, (e.g., DHA * + EPA + linoleic) (u)	0.98	3	ω-3 $\mathbf{CH_3}$–CH2–C=C	t (7.5)
Free cholesterol (u)	1.02	3	C19–$\mathbf{H_3}$	s
Esterified cholesterol (u)	1.04	3	C19–$\mathbf{H_3}$	s
FA, (Total fatty acyl chains) (u)	1.30	2	FA chain –$(\mathbf{CH_2})_n$–	m
FA, βH_2 (u)	1.62	2	βH_2 R–$\mathbf{CH_2}$–CH_2–CO–OR	m
FA (u)	2.02	2	–CH=CH–$\mathbf{CH_2}$–	m
FA, αH_2 (u)	2.35	2	αH_2–$\mathbf{CH_2}$–CO–OR	m
FA, (e.g., DHA*) (u)	2.41	4	α$\mathbf{H_2}$ and β$\mathbf{H_2}$ –CH=CH–$\mathbf{CH_2}$–$\mathbf{CH_2}$–CO–OR	m
FA, (e.g., linoleic) (u)	2.78	2	–CH=CH–$\mathbf{CH_2}$–$(CH=CH–CH_2–)_n$, *n* = 1	t (6.4)
FA, PUFA (e.g., DHA*) (u)	2.85	2	–CH=CH–$\mathbf{CH_2}$–$(CH=CH–CH_2–)_n$, *n* > 1	m
Sphingomyelin	3.37	9	–CH_2–N– $(\mathbf{CH_3})_3$	s
Phosphatidylcholine (u)	3.40	9	–CH_2–N– $(\mathbf{CH_3})_3$	s
Phosphatidylcholine	4.03	2	–$\mathbf{CH_2}$–N– $(CH_3)_3$	m
Triglycerides (u)	4.16	2	Glycerol (C1-$\mathbf{H^u}$) and (C3-$\mathbf{H^u}$)	dd (11.8, 6.1)
Triglycerides	4.31	2	Glycerol (C1-$\mathbf{H^d}$) and (C3-$\mathbf{H^d}$)	dd (12.1, 4.1)
Triglycerides	5.28	1	Glycerol (C2-**H**)	q
FA, MUFA and PUFA (e.g., DHA *) (u)	5.38	2	–**CH=CH**	m

FA: fatty acids; DHA: docosahexaenoic acid; EPA: eicosapentanoic acid; MUFA: monounsaturated fatty acids; PUFA: polyunsaturated fatty acids; Adapted from Vinaixa, M. et al. *J. Proteome Res*. **2010**, *9*, 2527–2538.

Table S2. Nuclear magnetic resonance (NMR) analysis of lipid extracts. Lipidomic phenotyping of not supplemented and supplemented cells with protocatechuic acid (PCA). Principal component analysis and canonical analysis (PCA-CA) discrimination accuracy and confusion matrix for the discrimination between "PCA" group and "no-PCA" after 6h (**left matrix**) and 24 h (**right matrix**). The discrimination accuracy for the comparison between the "PCA" group (PCA, PCA + docosahexaenoic acid (DHA)) and the "no-PCA" group (not supplemented (NS), propionic acid (PRO), DHA, DHA + PRO) was 53% after 6 h, and 57% after 24 h.

**6 h**	**PCA**	**No-PCA**	**24 h**	**PCA**	**No-PCA**
PCA	75	25	PCA	60	40
No-PCA	90	10	no-PCA	50	50
Discrimination accuracy: 53%			Discrimination accuracy: 57%		

Table S3. Nuclear magnetic resonance (NMR) analysis of lipid extracts. Lipidomic phenotyping of not supplemented and supplemented cells with propionic acid (PRO). Principal component analysis and canonical analysis (PCA-CA) discrimination accuracy and confusion matrix for the discrimination between "PRO" group and "no-PRO" group after 6h (**left matrix**) and 24 h (**right matrix**). The discrimination accuracy for the comparison between the "PRO" group (PRO, PRO + docosahexaenoic acid (DHA)) and the "no-PRO" group (not supplemented (NS), protocatechuic acid (PCA), DHA, DHA + PCA) was 50% after 6h, and 60% after 24 h.

**6h**	**PRO**	**No-PRO**	**24h**	**PRO**	**No-PRO**
PRO	65	35	PRO	65	35
No-PRO	80	20	no-PRO	50	50
Discrimination accuracy: 50%			Discrimination accuracy: 60%		

Table S4. Gas chromatography (GC) analysis of fatty acid composition. Fatty acid phenotyping of not supplemented and supplemented cells with protocatechuic acid (PCA). PCA-CA discrimination accuracy and confusion matrix for the discrimination between "PCA" group and "no-PCA" group after 6 h (**left matrix**) and 24 h (**right matrix**). The discrimination accuracy for the comparison between the "PCA" group (PCA, PCA+ docosahexaenoic acid (DHA)) and the "no-PCA" group (not supplemented (NS), propionic acid (PRO), DHA, DHA + PRO) was 46% after 6 h, and 52% after 24 h.

**6h**	**PCA**	**No-PCA**	**24h**	**PCA**	**No-PCA**
PCA	56	44	PCA	60	40
No-PCA	75	25	no-PCA	63	37
Discrimination accuracy: 46%			Discrimination accuracy: 52%		

Table S5. Gas chromatography (GC) analysis of fatty acid composition**.** Fatty acid phenotyping of not supplemented and supplemented cells with propionic acid (PRO). Principal component analysis and canonical analysis (PCA-CA) discrimination accuracy and confusion matrix for the discrimination between "PRO" group and "no-PRO" group after 6h (**left matrix**) and 24 h (**right matrix**). The discrimination accuracy for the comparison between the "PRO" group (PRO, docosahexaenoic acid (DHA) + PRO) and the "no- PRO" group (not supplemented (NS), protocatechuic acid (PCA), DHA, DHA + PCA) was 46% after 6h, and 52% after 24 h.

**6h**	**PRO**	**No-PRO**	**24h**	**PRO**	**No-PRO**
PRO	75	25	PRO	60	40
No-PRO	87	12	no-PRO	50	50
Discrimination accuracy: 54%			Discrimination accuracy: 56%		

Table S6. Nuclear magnetic resonance (NMR) analysis of water extracts. Metabolomic phenotyping of not supplemented and supplemented cells with protocatechuic acid (PCA)**. Principal component analysis and canonical analysis (**PCA-CA) discrimination accuracy and confusion matrix for the discrimination between "PCA" group and "no-PCA" group after 6h (**left matrix**) and 24 h (**right matrix**). The discrimination accuracy for the comparison between the "PCA" group (PCA, PCA + docosahexaenoic (DHA)) and the "no-PCA" group (not supplemented (NS), propionic acid (PRO), DHA, DHA + PRO) was 57% after 6h, and it slightly increased (67%) after 24 h. Accordingly, none of the metabolites analyzed showed significantly different levels in the "PCA" group with respect to the "no-PCA" group (Table 3).

**6h**	**PCA**	**No-PCA**	**24h**	**PCA**	**No-PCA**
PCA	70	30	PCA	83	17
No-PCA	70	30	no-PCA	67	33
Discrimination accuracy: 57%			Discrimination accuracy: 67%		

Table S7. Nuclear magnetic resonance (NMR) analysis of water extracts. Metabolomic phenotyping of not supplemented and supplemented cells with propionic acid (PRO). Principal component analysis and canonical analysis (PCA-CA) discrimination accuracy and confusion matrix for the discrimination between "PRO" group and "no-PRO" after 6h (**left matrix**) and 24 h (**right matrix**). The PCA-CA discrimination accuracy for the comparison between "PRO" group (PRO, docosahexaenoic (DHA) + PRO) and "no-PRO" group (not supplemented (NS), protocatechuic acid (PCA), DHA, DHA + PCA) after 24 h was around 85%. Creatine phosphate levels were lower in the "PRO" group with respect to "no-PRO" group after 6 h (p-value = 0.027), while no differences were detected after 24 h. A decrement of UMP levels in the "PRO" group was also monitored after 24 h (Table 3).

**6 h**	**PRO**	**No-PRO**	**24 h**	**PRO**	**No-PRO**
PRO	80	20	PRO	84	16
No-PRO	70	30	no-PRO	12	88
Discrimination accuracy: 63%			Discrimination accuracy: 85%		
